# Cardiovascular magnetic resonance assessment of ventricular morphology to investigate the mechanisms of heart failure associated with type 2 diabetes

**DOI:** 10.1186/1532-429X-17-S1-O82

**Published:** 2015-02-03

**Authors:** Peter P Swoboda, Adam K McDiarmid, Bara Erhayiem, Laura E Dobson, Tarique A Musa, David P Ripley, Pankaj Garg, Ramzi Ajjan, John P Greenwood, Sven Plein

**Affiliations:** 1 Multidisciplinary Cardiovascular Research Centre & Leeds Institute of Cardiovascular and Metabolic Medicine, University of Leeds, Leeds, UK

## Background

Type 2 diabetes mellitus (T2DM) is an independent risk factor for the development of heart failure. Data from clinical trials have demonstrated that patients with diabetic nephropathy have 2-3 times the risk of heart failure admission than those without nephropathy. Previous CMR studies have found frequent abnormalities in patients with T2DM including concentric left ventricular remodelling (increased relative wall mass), unrecognised myocardial infarction and raised extracellular volume (ECV). It is unknown which of these factors predispose patients to heart failure.

## Methods

80 asymptomatic patients with T2DM were recruited from primary care. All patients underwent CMR at 3.0T (Philips Achieva TX). Exclusion criteria included known cardiac disease, kidney disease (eGFR <30), uncontrolled hypertension, treatment with ACEi/ARB or insulin. 37 patients had persistent microalbuminuria (albumin creatinine ratio (ACR) (>2.5 for men, >3.5 for women on two or more measurements) and 43 had no documented microalbuminuria. Standard balanced steady state free precession cine images were acquired. T1 maps were acquired precontrast and 15 minutes after administration of 0.15mmol/kg gadobutrol. T1 maps were acquired using a an ECG triggered 5(3s)3 modified Look-Locker inversion recovery acquisition positioned at the short axis mid ventricular level. LV dimensions were calculated offline by summation of discs from a short-axis stack and myocardial extracellular volume calculated by measuring the pre and post contrast T1 times of myocardium and blood pool (cvi42 v4.1.3, Circle Cardiovascular Imaging Inc., Calgary, Canada).

## Results

There was no significant difference in patient characteristics including BMI, diabetes duration, HbA1c or blood pressure between the ACR +ve and ACR -ve patients (Table 1).

**Table 1:** Patient demographics shown as mean ± standard deviation unless otherwise stated.

	ACR -ve	ACR +ve	P value
Age	61.5 ± 8.7	58.50 ± 12.60	0.23

Male gender, n (%)	38 (88)	32 (86)	0.80

Body mass index	28.4 ± 3.7	29.60 ± 4.80	0.21

Duration of Diabetes, years	4.6 ± 3.7	5.40 ± 4.50	0.57

HbA1c, mmols/mol	59.4 ± 12.6	65.40 ± 19.70	0.27

Blood pressure, mmHg	130/72 ± 23/8	134/75 ± 17/10	0.37/0.10

Measurements of LV geometry including LVEDV, LVESV, EF, LV mass and relative wall mass (LV mass/EDV) were not significantly different between the two groups (Table 2). The incidence of myocardial infarction in ACR -ve cohort was 7/43 (16.3%) and was similar in the ACR +ve cohort 6/37 (16.2%). ECV was significantly higher in the ACR +ve group (27.2±4.2%) than in the ACR -ve group (25.1±2.8%, P=0.01).

**Table 2:** LV volumes and ECV shown as mean ± standard deviation unless otherwise stated.

	ACR -ve	ACR +ve	P value
End diastolic volume, ml	153.2 ± 32.4	151.8 ± 36.4	0.47

Ejection fraction, %	61.6 ± 5.3	59.7 ± 7.3	0.21

LV mass, g	95.4 ± 17.6	99.8 ± 25.2	0.52

Relative wall mass, g/ml	0.64 ± 0.13	0.67 ± 0.12	0.19

Precontrast T1 time, ms	1235.9 ± 40.3	1249.2 ± 66.6	0.46

ECV, %	25.1 ± 2.8	27.2 ± 4.2	0.01

## Conclusions

In this cohort of asymptomatic patients with T2DM there was no difference in concentric remodelling or the presence of myocardial infarction in patients with and without microalbuminuria. The only significant difference was a significantly elevated ECV suggesting that the increased risk of heart failure in T2DM with microalbuminuria may be mediated cardiac fibrosis and extracellular expansion.

## Funding

PPS (FS/12/88/29901) and SP (FS/10/62/28409) are funded by British Heart Foundation fellowships.

